# Undoing the unspeakable: researching racism in Swedish healthcare using a participatory process to build dialogue

**DOI:** 10.1186/s12961-019-0443-0

**Published:** 2019-04-23

**Authors:** Hannah Bradby, Suruchi Thapar-Björkert, Sarah Hamed, Beth Maina Ahlberg

**Affiliations:** 1 Department of Sociology, Uppsala University, Uppsala, Sweden; 2 Department of Government, Uppsala University, Uppsala, Sweden; 3 Skaraborg Institute and Department of Sociology, Uppsala University, Skövde and Uppsala, Sweden

**Keywords:** racism, healthcare, Sweden, participatory methods

## Abstract

**Background:**

Racism is difficult to discuss in the context of Swedish healthcare for various cultural and administrative reasons. Herein, we interpret the fragmentary nature of the evidence of racialising processes and the difficulty of reporting racist discrimination in terms of structural violence.

**Methods:**

In response to the unspeakable nature of racism in Swedish healthcare, we propose a phased participatory process to build a common vocabulary and grammar through a consultative framework involving healthcare providers and service users as well as policy-makers. These stakeholders will be involved in an educational intervention to facilitate discussion around and avoidance of racism in service provision.

**Discussion:**

Both the participatory process and outcomes of the process, e.g. educational interventions, will contribute to the social and political conversation about racism in healthcare settings. Creating new ways of discussing sensitive topics allows ameliorative actions to be taken, benefitting healthcare providers and users. The urgency of the project is underlined.

## Background

### Aim

The aim of this project is to create a discussion about racism in healthcare settings in Sweden, involving both healthcare providers and users, to inform and enable constructive interventions to reduce the damage of racism in healthcare interactions. This protocol sketches the limited evidence around racism in Swedish healthcare, frames it as a feature of structural violence and proposes a participatory intervention as crucial for beginning to address a long-standing institutionalised discrimination that damages patients, staff and the prospect of equal access to services.

Despite evidence of discrimination and inequality in health outcomes, racism is extraordinarily difficult to discuss in Swedish healthcare encounters. Therefore, the focus of our research is the fact that racism is apparent, yet unspeakable in the context of healthcare. We use the concept of structural violence to theoretically and empirically analyse how discrimination can be both widespread and hard to apprehend. This protocol takes healthcare as a context for structural violence and proposes a collaborative method to render racism a subject of discussion, thereby making it possible to cooperatively tackle racism by health service users and providers.

### Existing literature

The exceptionalism that constructs Sweden as gender equal, anti-racist and detached from a colonial past [1, 2], renders race, racism and racialising processes hard to conceptualise, let alone interrogate. Through the twentieth century, Sweden’s welfare state showed that the injustice of gendered and classed inequalities in mortality could be undone through a system of redistributive universal benefits [3]. Analysis of national register data shows health disparities between people born in Sweden and those born elsewhere across a range of conditions [4–7]. Yet, evidence of a migrant or minority health penalty [8] has received far less attention than class inequalities [9], despite equity and solidarity being key values for public health policy. The Swedish Public Health agency’s commitment to using analysis to improve equity in health^1^ notwithstanding, no data category exists for ethnic, cultural or racialised groups. Official attempts to grapple with racism in public services [10] have led neither to an agreed vocabulary nor a method for discussing, let alone tackling racism.

The experience or expectation of racism is a serious breach of the quality and accessibility of healthcare and represents a problem for both patients and professionals, yet there is minimal research of racism in the Swedish healthcare context [11]. Users of the Swedish healthcare system describe experiencing racism (Hamed et al., submitted), yet the Equality Ombudsman’s^2^ own reports suggest a significant level of under-reporting of racism and barriers to any reporting of discrimination.^3^ A small number of the cases reported to the Equality Ombudsman are upheld as constituting discrimination, but racism as a cause and consequence of discrimination is not mentioned.^4^

The Swedish health and social care work-force depends on foreign-born labour, whether skilled, semi-skilled or unskilled. Racialising gendered processes lead to patients’ discriminating against staff [12], as well as discrimination between staff [13] and between patients, which is even less researched. With little opportunity for healthcare staff from foreign backgrounds to discuss concerns about racism without jeopardising work-based relationships [13], the narrow cultural consensus neither accommodates newcomers’ expectations [14] nor makes space for their needs to be discussed [15]. Organisational routines, institutional categories [16] and practices that disadvantage particular groups [17, 18] create a context in which poor communication, involuntary compliance and disrespectful attitudes are experienced by service users and providers.

These contextual situations of discrimination, we argue, can be analysed through the theoretical lens of structural violence, which allows us to understand how the broader cultural and social institutions [19] shape risk of morbidity and mortality. To explain personal distress and disease, we need to embed individual biography in the larger matrix of culture, history and political economy [20]. Routinisation and normalisation of suffering coupled with ‘institutionalised social indifference’ renders the violence invisible – invisible not because it is hidden, but precisely the opposite – it is hardest to perceive because it is right before our eyes [21]. Structural inequities of power and knowledge embedded within medical culture not only nurture inequalities but also make ‘abnormal’ relationships appear ‘normal’ and thus invisible.

Structural violence is often embedded in long-standing ‘ubiquitous social structures’ that are normalised by stable institutions [22]: “[i]*n order to see violence, one must see the structures*” [23]. The total or partial withdrawal of health services from particular groups has been seen as a policy response to ongoing international migration (sometimes termed welfare chauvinism) and crises in healthcare provision for ageing populations have legitimated structural discrimination as a feature of universal healthcare systems. Where legal access to health services exists, the view that refugees and/or immigrants are less deserving than long-term residents has been termed ‘welfarism’ [24]. Thus, the potential development of an individual or group, which in our study is minority ethnic groups, is held back by the conditions of a specific relationship and in particular by the uneven distribution of power and resources [25]. Structural violence is done when people’s “*actual somatic and mental realizations are below their potential realizations*” [26].

Access to good quality healthcare can be obstructed by structural barriers compounded by individual-level discourtesies that individuals, however skilled and assertive, cannot easily address. In a project on healthcare access in diverse neighbourhoods [27], we interviewed two women of Somali origin in their 50s who had lived in Sweden for over 20 years, had learned Swedish, given birth, raised families and gained employment. They were friends and neighbours and both described neglectful healthcare when professionals did not listen to accounts of their serious symptoms. While they had both received good care at times, they felt that discrimination played a clear role, for instance, when trying to get an appointment. Access to professionals – both nurses and doctors in primary care – is restricted by gate-keepers with whom the patient has to negotiate via telephone. When speaking to these gate-keepers, the women felt discriminated against because their Swedish betrayed their foreign origins:

“*I also think that there is a difference as to how you would be treated if you are an immigrant and if you are Swedish. The person you speak to on the phone would decide whether or not to admit you and if you were Swedish you would probably be admitted… Since it’s a telephone conversation they can’t tell your race and they can judge by how you speak Swedish. You would definitely be (treated) different from a Swedish person*.”

It was not only in telephone-access to appointments that these women felt de-prioritised for care – antenatal care was denied to one when her labour had started but she was deemed not to have dilated sufficiently.

“*Sometime back I was pregnant and I went to the maternity/delivery hospital because of severe pains and they sent me back home, saying that I was not ready* [to give birth]. *The next day I stopped feeling the baby’s movement and the contractions stopped, so I was worried. My husband then took me for a walk but nothing happened, I still couldn’t feel the baby. I went to the hospital that evening again, this time I had excruciating pains and they told me that the baby is asleep and you are only 5 cm open. They called the doctor and they ran some tests and told me that they think the baby is either dead in my stomach or it is sleeping. So we will check to confirm and then see what to do, either to operate you when the baby is sleeping and or do a procedure if the baby is dead*.”

Her baby had died and she and her husband were, needless to state, devastated. The attitudes, conduct, speech and behaviour of the medical providers were facilitated by the wider matrix of embodied power, privilege and knowledge that infantilised and undermined the integrity of these women [24], although not necessarily intentionally. Referring to ‘multi-axial models of suffering’, Paul Farmer alerts us to the idea that simultaneous consideration of various ‘social axes’ (gender, race, ethnicity) is imperative in efforts to discern increased risk for extreme human suffering [20]. Social (deficient social security arrangements and absence of caring governance, racism, sexism) and economic forces (poverty) that constrain individual choice also constitute structural violence [20]. Structural violence reduces socially vulnerable minority populations to expendable non-persons, thus legitimising their medical inadequacies. Furthermore, these violations, we will argue, are not random misjudgements but symptoms of deeper, ‘pathologies of power’ [28], which determine who will suffer and who will be protected from harm.

One of the interviewees’ daughters had reported her high blood pressure during pregnancy and was also ignored:

“*My daughter almost lost her life there. She told them that she had high blood pressure but they didn’t hear her. They admitted her and by the morning it was at 200 and she almost died*.”

Healthcare professionals exercise the power of expert knowledge and experience over patients, whose vulnerability necessitates their surrender. This vulnerability makes it especially difficult for a patient and her family or informal carers to resist disparagement or disrespect from a professional. The specific social conditions in which a speaker is able to say something, and thereby to have some effect on others and on the world, is key to understanding the nuanced contingencies of racism as a process involving structural and individual elements that support structural violence in a healthcare context.

A man in his 20s, who was born in Chile but had migrated to Sweden as a child, described how his mother’s need was de-prioritised in an emergency clinic because of her appearance. He described how, after waiting for hours, his mother finally got medical attention only after she fainted. In common with the two women quoted above, this man spoke fluent Swedish, and was not prepared to make a formal complaint against the services, instead preferring to avoid the health services altogether. The difficulty of registering a complaint against discriminatory care experienced as racist means that no remedial quality-improvement action could be triggered and, in this respect, it differs from other failures of care. The violence embedded in the structure produces a “*paralysis and* powerless[ness] *among vulnerable populations forced into complicity with the very social forces that are poised, intentionally or not, to destroy them*” [21].

Where disrespect and/or discourtesy are part of a continuum of racist discrimination, there is an expectation of being further disregarded and, thus, no incentive to speak out. A woman of Sudanese origin described how migrants were regularly discriminated against in subtle ways that could not be proven. For example:

“*I felt that the Swedes get the established doctors while the foreigners get interns … it’s a feeling I got … but like I said, I don’t have proof…*”

Social interaction itself, coupled with silencing of weak voices reproduces the structures of domination. Thus, it is important to incorporate the voices of those who are silenced and those who keep silent about their suffering, whether they are service providers or users. The interview excerpts above are from service users and the voice of service providers and other relevant agencies is absent. To build on these patients’ stories of discrimination, we propose a participatory research process to facilitate dialogue and reflection among a broad range of stakeholders, including service providers and other agencies engaged with service support and policy-making as well as service users. This follows a strategy of researching ‘with’ not ‘on’ or ‘about’ people that focuses on practice, change and collaboration between practitioners and researchers [29]. Dialogue and reflection enables participants to question their shared assumptions in light of one another’s truths and build bridges to deal with the problem at hand. Participants are enabled to interrogate and unravel the social and institutional structures of domination and hierarchy in a participatory process below.

## Methods

We aim to use participatory methods, bringing together groups of healthcare users and groups of healthcare providers (groups which may, of course, be overlapping), to discuss racism in healthcare settings. The process depends on facilitating a discussion across and between various groups in order to find a common vocabulary and examples that can be agreed upon.

The perspective of communicative action theorists is premised on a critical reflective practice that is both ethical and creative but, most importantly, links knowledge to action. Knowledge that is generated and validated through social processes becomes institutionalised and no longer examined, evaluated or criticised. To challenge the embeddedness of routines and practices, we need emancipatory ways of ‘knowing’ that combine theory and praxis. These intersubjective practices lead to 'decolonialization of the lifeworld' and ‘communicative rationality’, in this case, to reveal rather than to conceal discussions on racism that potentially damage healthcare interactions [30, 31].

**Fig. 1:**
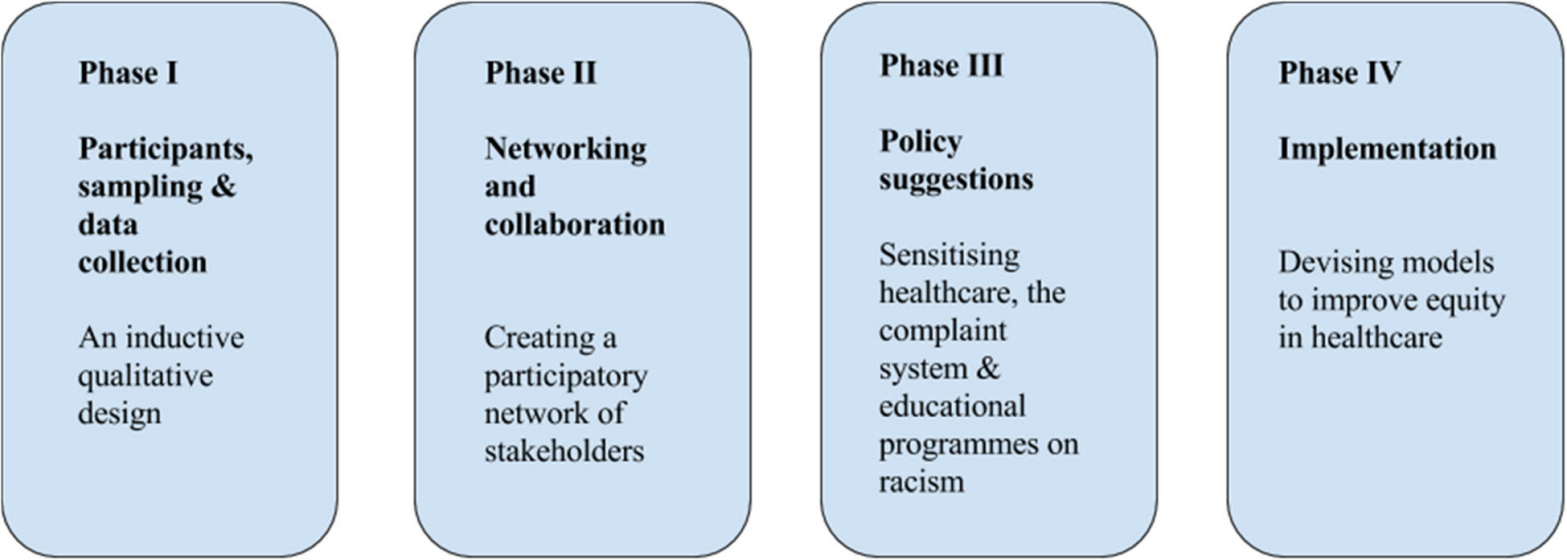
The four phases of the project

Our innovative and challenging process consists of four iterative phases (Fig. 1), as briefly described below.

Phase I explores and interrogates a range of people’s perceptions of racism in the context of diverse interactions and understanding how lived experiences are connected with meaning-making and knowledge production. This phase involves a critical analysis of the existing Swedish complaints system and a review of educational curricula in training programmes for health professionals, with respect to racism, focusing on anti-racist possibilities for patients and service providers, from multiple viewpoints. Interviews with healthcare users and providers and policy-makers will be conducted in urban and more rural settings.

This initial phase is a mapping of the problem area and a bridge to building the participatory process. Since racism is so unspeakable in Swedish healthcare settings, the research will be dependent on finding participants who are prepared to discuss these difficult and potentially threatening ideas. Interviewees will be recruited through civil society organisations and networks, including migrant associations and patient groups, as well as various healthcare centres and policy-makers from municipal and regional levels.

From experience with previous projects, we anticipate that a proportion of those we approach will decline to participate since being associated with the topic of racism carries the risk of being seen as a racist or as a victim of racism, neither of which are positive social roles. Reasons why people decline to participate will be of interest for the project, since this constitutes evidence of how racism is regarded by non-participants as well as participants. Given a likely high refusal rate, we anticipate the need to approach a wide range of potential participants, making use of all available contacts, from organisations and previous research, exploring all of our networks, both formal and informal. Trust must be established, which will mean being available to speak with people on their terms, in the time and place of their choice, whether singly or in groups.

Given the gendered difficulties of discussing racism in Sweden [32], the participatory intervention will only be effective and sustainable [33] if Phase II creates a collaborative network of key stakeholders from healthcare providers and patient associations. The material that emerges from the interviews will constitute the platform for organising dialogue and reflection in the networks of key stakeholders. Analysis of field notes, observations and transcriptions will be performed logically and inductively [34] and validated through discussion with the stakeholders.

From the analysis of field notes, observations and transcriptions, a number of vignettes will be prepared, which will set out descriptions of racism in healthcare settings from staff and patients’ perspectives. By presenting these vignettes to the collaborative network established in Phase II as the basis for further discussion, Phase III will generate policy strategies to promote equity in health services through a shared discussion of instances of racism that have been experienced in a network that has established participatory practice. It is anticipated that these could include guidelines for occupational routines in healthcare, tailored educational material for specific health professional programmes, simplification of the process of making complaints for both service users and providers, and an improved use of such complaints to feed back into developing practice and organisation.

The intervention will be evaluated through participatory discussions across the network using a peer-to-peer method. The policies developed through consultation will be implemented in a collaborating professional healthcare training programme to allow evaluation of the intervention and the introduction of changes where necessary.

Since this is a participatory process, dependent on the responses of stakeholders in the provision and use of healthcare services, the process cannot be prescribed in detail. The content of the material gathered in phase I, the response to the vignettes devised in phase II and the strategies for addressing racism generated in phase III are all guided by participants. The credibility of the process depends on including a range of stakeholders who will bring their own experiences and interpretations to bear on one another’s interpretations through discussion – thus the centrality of establishing a participative network, since participants willingness to challenge one another’s interpretations of vignettes is the guarantee of arriving at shared understandings of how racism plays out in healthcare settings.

Finally, Phase IV involves implementation, whereby the outcomes of the strategies are evaluated and areas for improvements are identified. Using a University professional healthcare training programme, an educational intervention will be piloted, together with stakeholders, to sensitise healthcare providers against racism.

## Discussion

All stages of the work will be incorporated in a final analysis to engage a range of parties in an exercise of shared knowledge production. The initial review of literature and policy, the description of organisations and processes, and qualitative methods, including discourse and thematic analysis, will all be used to organise and classify material. This represents an extension of our previous work [18] in analysing how racialised and gendered practices play out in institutional settings despite broad policy goals that may be progressive. The lack of an official discourse about racism in Sweden (even from the ombudsman responsible for discrimination in care) and the rhetoric of exceptionalism, makes the way that subtle discourtesies (or micro-aggressions) accumulate alongside outright discrimination (structural or individual), extremely hard to describe and map. Only by adopting a consultative framework, leading to a jointly-owned co-sponsored intervention, will it be possible to speak about racism and therefore start undoing it. This work is necessary and overdue given the insistence on the absence of racism from Swedish public services.

## Footnotes

1
https://www.folkhalsomyndigheten.se/the-public-health-agency-of-sweden/about-us/our-mission/

2 *Diskrimineringsombudsmannen* or Equality Ombudsman is the statutory body that handles complaints about health services as part of a legal duty to promote equality and counteract discrimination.

3
http://www.do.se/om-diskriminering/publikationer/upplevelser-av-diskriminering/

4 http://www.do.se/om-diskriminering/publikationer/ratten-till-sjukvard-pa-lika-villkor/ The Ombudsman asserts that healthcare should not take “*irrelevant account*” (*ovidkommande hänsyn*) of a range of factors: ‘*kön, könsidentitet och könsuttryck, etnisk tillhörighet, religion eller annan trosuppfattning, funktionsnedsättning, sexuell läggning eller ålder’*. This translates as “*gender, gender identity and gender expression, ethnic affiliation, religion or other beliefs, disability, sexual orientation or age*”. There is no mention of racialised identity or of racism as a possible basis for discrimination.
